# Acute Stanford Type A Aortic Dissection: A Review of Risk Factors and Outcomes

**DOI:** 10.7759/cureus.36301

**Published:** 2023-03-17

**Authors:** Michael Chukwu, Paghunda Ehsan, Rawia N Aburumman, Shivani Ishwarya Muthanna, Swathi Radhakrishnan Menon, Vruti Vithani, Bansi Sutariya, Diana M Montenegro, Lubna Mohammed

**Affiliations:** 1 General Surgery, California Institute of Behavioral Neurosciences & Psychology, Fairfield, USA; 2 General Surgery, Pilgrim Hospital, Boston, GBR; 3 Research, California Institute of Behavioral Neurosciences & Psychology, Fairfield, USA; 4 Internal Medicine, Hayatabad Medical Complex, Peshawar, PAK; 5 Internal Medicine, Lady Reading Hospital, Peshawar, PAK; 6 Internal Medicine, Mu'tah University, Amman, JOR; 7 Research and Development, California Institute of Behavioral Neurosciences & Psychology, Fairfield, USA; 8 Internal Medicine, Vydehi Institute of Medical Sciences and Research Centre, Bangalore, IND; 9 Pediatrics, California Institute of Behavioral Neurosciences & Psychology, Fairfield, USA; 10 Internal Medicine, California Institute of Behavioral Neurosciences & Psychology, Fairfield, USA; 11 General Medicine, Government Medical College, Surat, IND

**Keywords:** outcomes, risk factors, acute type a dissection, stanford type a acute aortic dissection, aorta repair, complications of aortic dissection

## Abstract

Acute aortic dissection (AAD) can be said to be a relatively uncommon emergency with fatal outcomes mainly due to delayed/missed diagnosis and treatment. Its ability to masquerade as other emergencies like acute coronary syndrome and pulmonary embolism makes the prognosis unfavorable in a significant proportion of patients. Patients have been seen to present to the accident and emergency department or outpatient setting with typical or atypical symptoms as we will discuss in this article. We have focused on indicators for risk and prognosis of acute Stanford type A aortic dissection in this traditional review. It is well known that despite recent developments and improvements in treatment modalities, AAD is still associated with a significant mortality rate and postoperative complications.

## Introduction and background

The aorta is the main blood vessel extending from the left heart to the other body organs and tissues. Dissection of the aorta typically occurs when the aortic media separates from the intima; this results in an accumulation of blood in this space which is different from the original lumen of the aorta. If this progresses, it can extend proximally or distally, affecting the branches of the aorta and leading to the various clinical presentations of this condition [1]. The Stanford and DeBakey classifications are the popularly used means of classifying type A aortic dissection (AD), established in 1970. Type A AD includes the ascending aorta, arch, and descending thoracic aorta, while type B AD involves dissections that commence in the descending aorta, regardless of any retrograde involvement of the aortic arch. Among cardiothoracic surgical emergencies, acute type A AD is deemed a highly lethal condition [2]. 70-75% of acute aortic dissections (AADs) are Stanford type A. AAD makes up more than 80% of acute aortic syndromes, including atherosclerotic aortic ulcer and intra-aortic hematoma [3]. 

We reviewed the risk factors and outcomes of acute type A AD and multispecialty care required for good patient outcomes. Over the years, presentation and examination findings remain relatively the same, with few atypical presentations. Fortunately, using computed tomography (CT) aortogram has improved the early diagnosis of type A AD and, consequently, the outcomes. In terms of demographics, Stanford type A AD generally affects the elderly. This condition can present like a heart attack where patients may present to the emergency room with tearing central chest and mid-back pain; sometimes, it can be described as a ripping or shearing pain, radiating to the neck or upper back. It can also mimic stroke, where patients would present with hemiparesis; however, it can present with different vague symptoms leading to misdiagnosis. Late presentation/treatment to health care facilities is mainly exacerbated by delays and limited access to diagnostic tests such as CT scans. Management of acute Stanford type A AD remains a challenge in surgical practice as it is a time-critical condition. However, modalities have significantly improved in recent times [4]. The outcome is primarily influenced by several factors, including time of presentation, prompt surgical repair, complications, preceding co-morbidities, and age of the patient. Possible complications include acute-onset cardiac dysfunction, cardiac tamponade, impaired perfusion of the coronary arteries, aortic valve incompetence, and end-organ damage; as a result, there can be a sudden hemodynamic deterioration or drop in clinical status/stability [1]. Unfortunately, even with timed and early surgical treatment, the mortality rate of this condition is higher than 20% [3].

Type A AD occasionally presents with deficits in radial pulse and blood pressure; there are significant cases where this is absent. Therefore, a high index of suspicion is paramount to the diagnosis and early treatment [5]. There have been reported cases of type A AD with symptoms of only headache and a history of migraine [6]. In our literature, we dwelled on Stanford type A AD which involves dissection of the ascending aorta before the branch-off point of the brachiocephalic trunk with or without distal progression to involving the descending aorta. About a third of most cases occur in patients above 75 years of age, making age a solid predisposing factor to this fatal condition. Over the years, several cases of this condition have been reported, but it remains a significantly rare but difficult surgical emergency. The current incidence is 8.7 per 100,000 patient years. However, it is being extrapolated that there will be a consequential increase in the overall incidence of this condition by 57% by 2050 due to a general increase in life expectancy in the populace. Potential risk factors include congenital causes, lifestyle and cardiovascular factors, and inflammatory, infectious, trauma, and iatrogenic causes, as shown in Table 1.

**Table 1:** Risk factors

Risk factors for acute aortic dissection	
Congenital causes	Marfans syndrome, Ehlers-Danlos syndrome, Turners syndrome
Lifestyle and cardiovascular risk factors	Family history, advanced age, smoking, cocaine
Inflammatory and infectious causes	Large vessel vasculitis, tertiary syphilis, tuberculosis
Trauma	Chest trauma and injuries.
Iatrogenic	Cardiac surgical procedure.

Regarding treatment and outcome, the choice of surgical procedure or treatment depends on the presentation, exact pathology site, and co-morbidities. The approach should be tailored adequately to suit the patient's needs on an individualized scale. The common surgical management of acute type A AD is an open procedure, though not all patients are eligible for these due to varying individual risks; about 25% of patients may not benefit from this procedure [7]. The overall outcome is also dependent on age, as the results of surgical therapy for this condition in the elderly have been controversial and burdened with a high surgical mortality rate [8]. Recently, there has been a surge in complex and sophisticated surgical therapeutic options. However, this lethal condition has remained a stubborn challenge for heart surgeons worldwide [9].

The overall outcome of acute type A AD is increasing regardless of the significant morbidity and mortality rates. Patients who have conservative surgical approaches have been shown to reduce morbidity to some degree potentially. Ongoing research and work on better measures are undertaken to minimize complications and prevent these events, mainly by long-term follow-up of high-risk patients and institution of planned prophylactic repair in selected cases [1]. It is noteworthy knowing that with the improvement in the standard of care in most surgical settings and recent enhancements in examination and investigation techniques and resuscitation modalities, there is a possibility of improved outcomes. This has made an early diagnosis, systematic analysis of risk factors on an individualized basis, development of special aortic teams, and regionalization of management key for future reduction in complications and poor outcomes [3].

## Review

Methods and results

A review of the literature was performed electronically using PubMed, Google Scholar, and The Cochrane Library using search keywords and medical subject heading terms such as "Stanford type A,' 'acute aortic dissection,' 'risk factors' 'outcomes,' 'arch replacement,' 'aortic replacement,' 'arch repair,' 'acute type A dissection', and synonyms. Search results over the last five years yielded 331 articles (PubMed), 88 articles (Google Scholar: 2018-2022), and 52 articles (The Cochrane Library). We were able to isolate more than 50% of the articles at first screening as they were not fit for the research and focused on a total of 26 articles for this review. The literature review was limited to English, within the last five years, and no age restriction was placed on the searches. A literature review showed a significant variation in patients' clinical symptoms having AAD. Studies have shown that advanced age, history of cardiac procedures, cardiopulmonary resuscitation before surgery, blood transfusion, and renal impairment after surgery are significant independent factors posing a risk for mortality in AD [10].

Discussion

Stanford type A AD remains a lethal emergency despite advances in the last few decades. The International Registry of Acute Aortic Dissection (IRAD) states that the mortality for surgically managed cases is around 26% [11]. In terms of predictive value, a study found that the history of hypertension is a weak predictor of AD, connective tissue disorders were poor, and the history of coronary vascular disease did not predict AAD, as shown in Table 2 [12].

**Table 2:** Comparison of possible predictive factors for acute aortic dissection

Possible Predictive factor	Sample number(n)	Specificity	Positive likelihood ratio (LR+)	Sensitivity	Negative likelihood ratio (LR-)
History of hypertension	7	37%-64.1%	1-1.53, I2=47%	56.2%-80%	0.61-1, I2=84%
Connective tissue disorders	3	84%-97%	0.09-16.54, I2=84%	5%	1.11, 95% CI=0.67-1.83, I2=0%
History of coronary vascular disease	1	12%	1.29, 95% CI= 1.14-1.45	68.1%	0.39, 95% CI

About two-thirds of AAD cases are type A, and 67% of them are men with an average age of 63 years. Hypertension is stated to be the most common risk factor (76%). Patients <40 years are more likely to have Marfan syndrome or bicuspid AD as a risk factor compared to older individuals, where hypertension and atherosclerosis are more likely. A study showed that nearly two-thirds of patients have an aortic luminal diameter of less than 5.5 cm; hence, smaller aortic diameters do not rule out AD. This is because the etiology of AD varies among individuals. There is little knowledge about the size of the aorta just before the dissection occurs [13]. Due to modern and more in-depth studies on AD, there have been significant considerations of expansion rate, age at diagnosis, and diameter of the aorta when the chance of dissection increases; all of these, including family history, strongly affect the outcome [14-16]. Research has shown that about 6.4-11% of AAD are painless; in these cases, patients may present with relatively rare symptoms such as loss of consciousness, peripheral weakness, paralysis of limbs, or abdominal discomfort.

It is conventionally accepted that AAD has increasing mortality with a delay in diagnosis at a rate of about 1-2% per hour. Risk factors are good markers for early diagnosis of AAD, combined with the characterization of clinical symptoms such as pain and physical examination. The average time taken from hospital presentation to diagnosis is about 4.3 hours. Interestingly, AAD, just like other cardiac and vascular diseases, has a chronobiological pattern, as it has a higher frequency of occurrence between 6 am and 12 pm, with the highest being between 8 am and 9 am (P<0.001) [13]. The most commonly identified symptom in acute type A AD is chest pain (79%) as against back pain which is the commonest for type B AD (64%). There is a higher chance of mortality in patients with atypical symptoms, for example, acute abdominal pain, as there could be a delay in diagnosis. There is a higher incidence of hypertension in type B than in type A (70% vs. 36%) [13].

Post-traumatic stress disorder can occur because of mental and physical health consequences of type A AAD and its repair on the patient. Poor outcomes can include hemopericardium and hemothorax, and the higher incidence of pre-operative cardiogenic shock may theoretically increase mortality. This may inform the need for conservative management in cardiogenic shock or tamponade patients. It has been shown that 35% and 70% of proximal and distal AAD have elevated blood pressure. Therefore, blood pressure measurement should be done bilaterally in the upper or lower limbs, and a comparison made for the deficit. However, contrary to the emphasis, there is less than 20% of cases presented with bilateral blood pressure deficit. Cardiac auscultation may reveal a murmur of aortic insufficiency in about half of the cases [17]. Surprisingly, many patients can have normal D-dimer and blood pressure at presentation. Many patients present with no clear biochemical or radiological evidence. The survival phases/period for AAD is shown in Table 3.

**Table 3:** Survival phases/periods

Time	Definition
Hyperacute	Symptom onset to 24 hours
Acute	2-7 days
Subacute	8-30 days
Chronic	>30 days

Going through the survival curve, there is a progressive decline in the general survival rates [13]. Eighty-six percent of type A AAD are often managed by surgery, which has recently increased compared to three decades ago when it was about 70%. Fifty-seven percent of those managed medically have a poor outcome. The in-hospital mortality rate is around 22% compared to the 31% previously noted [13]. To improve the chances of a favorable outcome, the initial step is to have a high index of suspicion while identifying significant risk factors through good history taking [17]. Considerable delay in diagnosis can also be influenced by the choice of investigation, for example, when magnetic resonance imaging or aortogram is done instead of a CT or echocardiography [18]. Atypical symptoms may inform this at the initial presentation to the emergency department. Unlike acute coronary syndrome and pulmonary embolism, AAD lacks specific biomarkers, making diagnosis difficult from laboratory findings alone. Given this fact, using a standard diagnostic approach, regular AAD training, retraining programs, and ongoing trauma and emergency department educational sessions are not vital in reducing mortality and morbidity [19].

Major complications associated with type A AAD are hypotension, shock, recurrent pain, refractory hypertension, pericardial effusion, cardiac tamponade, periaortic hematoma, visceral ischemia, peripheral ischemia, renal insufficiency, mesenteric malperfusion, acute limb ischemia, and brain injury [20]. Regardless of the final means of surgical or non-surgical, "anti-impulse" treatment is indicated to reduce aortic wall stress. Beta-blockers are the recognized first-line medical therapy, intending to achieve a pulse rate of less than 60 beats per minute (bpm). However, in some cases where aortic valve insufficiency is a complication, the pulse rate may need to be maintained at a high rate, hence achieving compensatory tachycardia, which helps achieve desired cardiac output [21]. Alongside this, hypertension control is essential in all patients regardless of the type of AAD; one of the reasons is, beta-blockers are the first line as they can control blood pressure and heart rate. They have also been shown to protect the heart muscles from ischemic changes. The recommendation is the maintenance of systolic blood pressure somewhere between 100 millimeters of mercury (mmHg) and 120 mmHg, in cases where beta-blockers cannot be used; second-line agents include calcium channel blockers or nitroglycerin [22]. The primary goal of surgical therapy in AAD is to avoid rupture of the aorta and cardiac tamponade and relieve aortic insufficiency [23]. The decision on the need for repair depends on a precise analysis of the benefits and risks of surgical repair. However, the general recommendation is surgical management for a diameter of five or 5.5 cm and above [24].

For type A AAD, surgical results are still unsatisfactory; the overall early mortality rate is around 9-25%. In terms of prognosis and long-term follow-up, the overall survival for all patients undergoing aortic surgery in one study was 84.7% after a year, 78.3% after three years, and 72.5% after five years. Survival for patients free of hospital death was 93.4% at one year, 86.3% at three years, and 79.9% at five years. The initial risk of complication (death, paraplegia, or stroke) for type A AD repair (including elective, urgent, and emergent) was 10.5%. Compared to elective operations, the chance of permanent damage is more than six-fold higher if the procedure is performed urgently or as an emergency. Increasing age raised the chances of suffering a significant complication. When compared with the age group 55 years or less, the age group 55 to 64 years had a threefold higher likelihood of adverse events, and among the 65 and older group, the odds were more than threefold higher [25].

The overall outcome post-op is significantly better than isolated medical management. With newer modalities, some cardiac surgical centers have safer/better outcomes with mortality rates as low as 3% in referral facilities [25,26]. The diameter, though important, should not be the sole parameter in decision-making; other factors, including clinical state, should also be considered. Regarding the prevention of AAD, measures should be put in place to ensure efficient identification of at-risk individuals, detailed family history, prophylactic procedures, adequate control of hypertension, and pharmacotherapy. Therefore, a careful analysis of genetic and environmental factors through radiological and genetic tests will go a long way in reducing the overall risk associated with AAD [19].

## Conclusions

Type A AAD remains a cardiovascular emergency with high morbidity and mortality in late presentation. Although treatable, poorly managed premorbid hypertension is the commonest risk factor. Our review has shown that the overall reason for high mortality in AAD is multifactorial, mainly age, history of heart surgeries, cardiopulmonary resuscitation, and post-operative acute renal injury. Based on recent findings, data collection, and regular audits will improve awareness, reduce delays in diagnosis and treatment, and further elucidate the risk factors and treatment outcomes. Immediate and appropriate management is essential to attain the best results. However, the ideal surgical approach for various situations is controversial. Medical innovations toward AAD have shown to be geared toward preventing modifiable risk factors and defining barriers to care.

We look forward to newer technologies in improving our clinical knowledge of the anatomical and physiological properties of the aorta wall, as this will further inform the development of safer and more effective guidelines for managing AAD.
